# Unveiling Carbon Dioxide and Ethanol Diffusion in Carbonated Water-Ethanol Mixtures by Molecular Dynamics Simulations

**DOI:** 10.3390/molecules26061711

**Published:** 2021-03-19

**Authors:** Mohamed Ahmed Khaireh, Marie Angot, Clara Cilindre, Gérard Liger-Belair, David A. Bonhommeau

**Affiliations:** Université de Reims Champagne-Ardenne, CNRS, GSMA UMR 7331, 51097 Reims, France; mohamed.ahmed-khaireh@univ-reims.fr (M.A.K.); marie.angot@univ-reims.fr (M.A.); clara.cilindre@univ-reims.fr (C.C.)

**Keywords:** ethanol, carbon dioxide, diffusion, molecular dynamics, viscometry

## Abstract

The diffusion of carbon dioxide (CO${}_{2}$) and ethanol (EtOH) is a fundamental transport process behind the formation and growth of CO${}_{2}$ bubbles in sparkling beverages and the release of organoleptic compounds at the liquid free surface. In the present study, CO${}_{2}$ and EtOH diffusion coefficients are computed from molecular dynamics (MD) simulations and compared with experimental values derived from the Stokes-Einstein (SE) relation on the basis of viscometry experiments and hydrodynamic radii deduced from former nuclear magnetic resonance (NMR) measurements. These diffusion coefficients steadily increase with temperature and decrease as the concentration of ethanol rises. The agreement between theory and experiment is suitable for CO${}_{2}$. Theoretical EtOH diffusion coefficients tend to overestimate slightly experimental values, although the agreement can be improved by changing the hydrodynamic radius used to evaluate experimental diffusion coefficients. This apparent disagreement should not rely on limitations of the MD simulations nor on the approximations made to evaluate theoretical diffusion coefficients. Improvement of the molecular models, as well as additional NMR measurements on sparkling beverages at several temperatures and ethanol concentrations, would help solve this issue.

## 1. Introduction

Sparkling alcoholic and non-alcoholic beverages are massively consumed since decades all over the world [1,2,3]. Dissolved carbon dioxide (CO${}_{2}$) is obviously the gaseous species responsible for the sparkle in every sparkling beverage. In premium sparkling wines, for example, dissolved CO${}_{2}$ results from a second in-bottle fermentation process promoted by adding yeasts and sugar in a still base wine stored in thick-walled glass bottles hermetically sealed with a crown cap or a cork stopper [4]. In bottled or canned beers, dissolved CO${}_{2}$ is also the result of a natural fermentation process [5,6]. In carbonated soft drinks (and in some cheaper sparkling wines and sparkling waters) carbonation is rather undertaken by forcing exogenous gas-phase CO${}_{2}$ to dissolve into still soft drinks, and by conditioning them in cans or in bottles most often sealed with crown or screw caps [1].

The capacity of gas-phase CO${}_{2}$ to get dissolved in a liquid phase is ruled by Henry’s law, which states that the concentration of dissolved CO${}_{2}$ found in the liquid phase remains proportional to the partial pressure of gas-phase CO${}_{2}$ found in the sealed bottle or can [7]. From the industrial angle, the level of dissolved CO${}_{2}$ in the beverage is a parameter of importance, because it is responsible for the very much sought-after bubbling process. Under standard tasting conditions, the level of dissolved CO${}_{2}$ found in a sparkling beverage directly impacts various sensory properties, such as the growth rate of ascending bubbles [8,9], the overall number and size of bubbles likely to form in a glass [10,11], the release of aromas through bursting bubbles [12,13,14], the kinetics of the degassing process [15,16,17,18,19], and the very characteristic tingling sensation in mouth [20,21,22]. Suffice to say that the presence of dissolved CO${}_{2}$ strongly modifies the organoleptic properties of the sparkling beverage.

**Figure 1:**
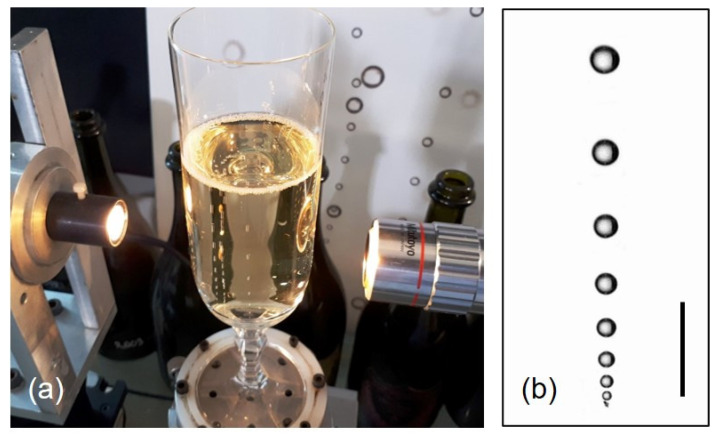
High-speed video imaging device aimed at filming ascending bubbles in glasses poured with sparkling wines (**a**), with a micrograph showing CO${}_{2}$ bubbles rising in-line and growing by diffusion along the side of the glass (**b**) (bar = 1 mm). (photographs by G. Liger-Belair).

In the past two decades, the chemical physics behind bubble dynamics has been thoroughly investigated in champagnes [3,8,23], beers [6,8,10,24], carbonated soft drinks [25,26], and sparkling waters [27,28,29], both experimentally and theoretically. In addition to the level of dissolved CO${}_{2}$ responsible for modifying the overall sensory properties of carbonated beverages, as described above, another key parameter of CO${}_{2}$ was pointed out as being significantly involved in the bubbling process, as well as in the degassing process, under standard tasting conditions. This is the bulk diffusion coefficient of dissolved CO${}_{2}$ in the liquid phase [30]. Indeed, the frequency of bubble nucleation in a glass, as well as the growth rate of ascending bubbles, both increase with the diffusion coefficient of dissolved CO${}_{2}$ [23]. As exemplified in Figure 1, it was determined that small bubbles rising in-line in a glass of sparkling wine grow by diffusion, with a theoretical growth rate *k* expressed by the following relationship [9]:

(1) $$k=\frac{dr}{dt}\approx 0.63\frac{RT}{P}D^{2/3}{\left(\frac{2\rho g}{9\eta }\right)}^{1/3}\left(c_{L}-c_{0}\right),$$

where *r* is the bubble radius (in m), *t* is the time (in s), *R* is the ideal gas constant ($\approx 8.314\;J\;K^{-1}\;{\mathrm{mol}}^{-1}$), *T* is the liquid phase temperature (in K), *P* is the partial pressure of CO${}_{2}$ within the bubble (close to 10${}^{5}$ Pa), *D* is the bulk diffusion coefficient of CO${}_{2}$ in the liquid phase (in m${}^{2}$ s${}^{-1}$), ρ is the liquid density (in kg m${}^{-3}$), *g* is the acceleration of gravity (≈$9.8\;m\;s^{-2}$), η is the liquid dynamic viscosity (in Pa s), $c_{L}$ is the bulk concentration of dissolved CO${}_{2}$ in the liquid phase (in mol m${}^{-3}$), and $c_{0}$ is the concentration of dissolved CO${}_{2}$ close to the bubble’s interface in Henry’s equilibrium with gas-phase CO${}_{2}$ in the bubble (in mol m${}^{-3}$). The previous equation, indeed valid for small CO${}_{2}$ bubbles rising in a carbonated beverage, reveals the crucial role of the bulk diffusion coefficient of CO${}_{2}$ in the bubble growth.

Moreover, in sparkling alcoholic beverages, the rate at which ethanol (EtOH) evaporates (and, therefore, also influences the sensory properties of the beverages) depends on the diffusion coefficient of EtOH [13,14,31]. Actually, these crucial transport properties of both CO${}_{2}$ and EtOH involved in sparkling beverage tasting were found to strongly increase with the temperature of the liquid phase, as thoroughly described through classical molecular dynamics (MD) and nuclear magnetic resonance (NMR) spectroscopy in brut-labeled Champagne wines (i.e., champagnes with concentrations of sugars < 12 g L${}^{-1}$) [32,33,34]. Otherwise, from the lowest-alcohol beers and the sweet ciders to the highest alcohol beers and sparkling wines, the sparkling alcoholic beverage segment is characterized by a very wide range of ethanol levels (from close to 0 to almost 15% ethanol by volume). Yet, by strongly modifying the viscosity of water-ethanol mixtures and commercial beverages [35,36,37], no doubt that the concentration of ethanol should significantly modify the subsequent transport properties of every species found in the water-ethanol mixture, including the respective diffusion coefficients of both CO${}_{2}$ and EtOH.

In this article, the diffusion coefficients of CO${}_{2}$ and EtOH are evaluated by MD simulations as a function of the volumetric alcoholic title at three temperatures relevant for applications on carbonated alcoholic beverages. They are compared with experimental values derived from the Stokes-Einstein (SE) relation on the basis of viscosity measurements in the same section. The results are presented and compared with data from the literature in Section 2. The influence of the hydrodynamic radius definition on the comparison between theory and experiment and possible improvements of the theoretical approach are discussed in Section 3, before proposing openings to the present study in Section 4. Details on MD simulations and experimental measurements are provided in Section 5 together with the strategy followed to evaluate CO${}_{2}$ and EtOH diffusion coefficients.

## 2. Results

**Figure 2:**
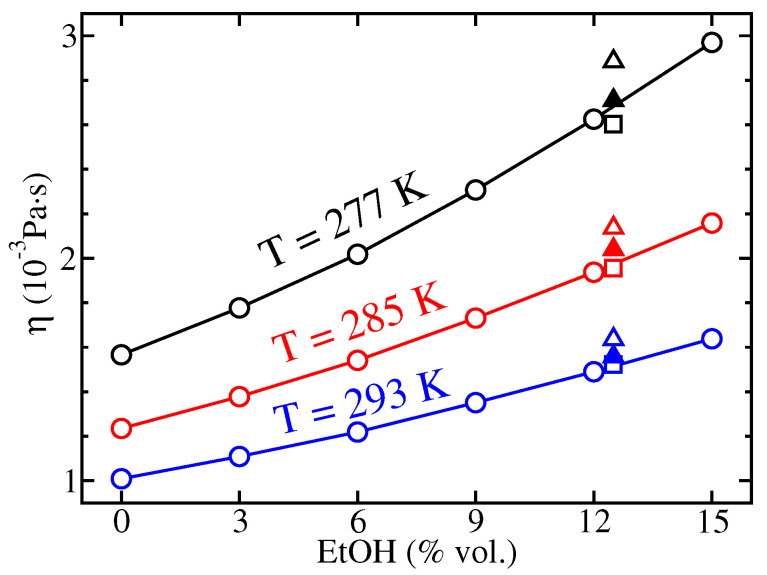
Experimental viscosities of hydroalcoholic solutions at *T* = 277 K (black circles), *T* = 285 K (red circles), and *T* = 293 K (blue circles). Viscosities from the literature obtained for hydroalcoholic solutions (open squares) and brut-labeled champagnes (triangles) are also indicated [33]. Data on brut-labeled champagnes are derived from viscometry (open triangles) and an Arrhenius law (closed triangles) [33,39].

Experimental viscosities are depicted in Figure 2 as a function of the EtOH level expressed as percentage by volume at the three temperatures of interest. As expected, they increase regularly as the alcoholic degree increases and the temperature decreases. These results are in agreement with former measurements carried out on carbonated hydroalcoholic solutions with ethanol concentrations representative of brut-labeled champagnes (∼12.5%vol.). They are slightly smaller than viscosities expected in champagnes because of the broad variety of species other than water, CO${}_{2}$, and EtOH, contained in these beverages like glycerol, lactic and tartaric acids, and sugars [38].

**Figure 3:**
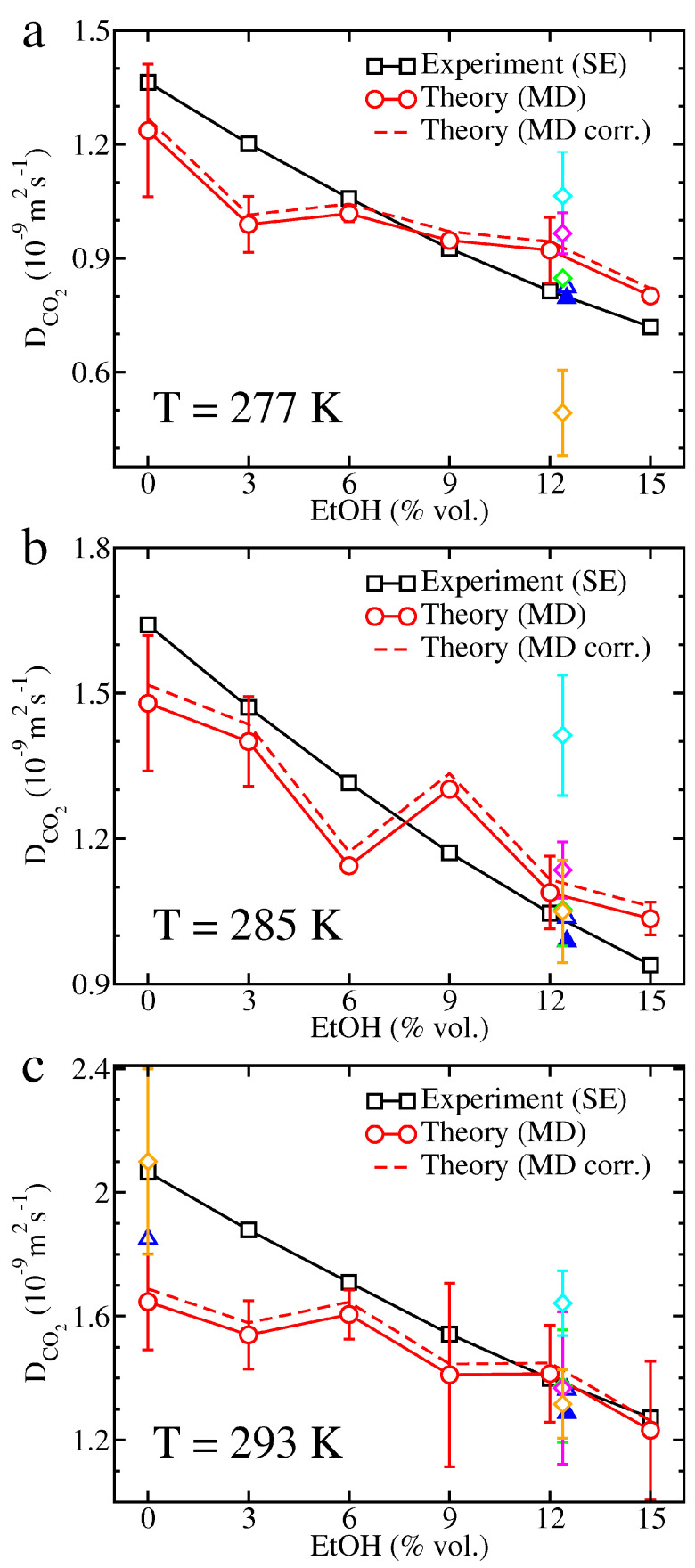
Experimental and theoretical CO${}_{2}$ diffusion coefficients in carbonated hydroalcoholic solutions at (**a**) *T* = 277 K, (**b**) *T* = 285 K, and (**c**) *T* = 293 K. Experimental values (black squares) derived from the Stokes-Einstein (SE) relation and theoretical values deduced from molecular dynamics (MD) simulations (red circles) are reported together with theoretical diffusion coefficients corrected for system-size dependence (red dashed curve). Data from the literature are also indicated: MD simulations on carbonated hydroalcoholic solutions (ethanol (EtOH) at 12.4% vol.) where water is described by the OPC (Optimal Point Charge) model (green diamond) [34], TIP4P/2005 (Transferable Intermolecular Potential with 4 Points/2005) model (magenta diamond) [34], SPC/E (Extended Simple Point Charge) model (orange diamond) [33,42], and TIP5P (Transferable Intermolecular Potential with 5 Points) model (cyan diamond) [33]; ${}^{13}$C nuclear magnetic resonance (NMR) measurements on carbonated hydroalcoholic mixtures [33] or fizzy water [30] (blue open triangles) and brut-labeled Champagnes (blue closed triangles) [33].

CO${}_{2}$ diffusion coefficients derived from MD simulations and experimental viscosities determined by applying the Stokes-Einstein (SE) relation are reported in Figure 3. The agreement between theory and experiment is generally satisfactory at all temperatures and alcoholic degrees provided that statistical uncertainties are taken into account. Correcting theoretical diffusion coefficients for their possible system-size dependence only yields a slight increase of diffusion coefficients that does not alter the overall agreement with experimental data. Strikingly, the worst agreement is obtained at T=293K and low concentrations of ethanol, whereas the OPC (Optimal Point Charge) water model reproduces accurately water self-diffusivity at such a temperature [40], a behavior that we checked in simulation boxes only filled with water molecules. However, it is worth noting that the CO${}_{2}$ diffusion coefficient in fizzy water was found to be $1.85\times 10^{-9}\;m^{2}\;s^{-1}$ [30] and previous MD simulations modeling CO${}_{2}$ diffusion in SPC/E (Extended Simple Point Charge) water [41] lead to CO${}_{2}$ diffusion coefficients of about $2.1\times 10^{-9}\;m^{2}\;s^{-1}$ with relatively large uncertainties: in het Panhuis et al. [42] and Perret et al. [32] respectively found values of $\left(2.1\pm 0.3\right)\times 10^{-9}\;m^{2}\;s^{-1}$ and $\left(2.11\pm 0.14\right)\times 10^{-9}\;m^{2}\;s^{-1}$ when computing the MSD of diffusing CO${}_{2}$ molecules, and, in het Panhuis et al. [42] obtained values of $\left(2.1\pm 0.08\right)\times 10^{-9}\;m^{2}\;s^{-1}$ and $\left(1.8\pm 1.33\right)\times 10^{-9}\;m^{2}\;s^{-1}$ after calculating the velocity and force autocorrelation functions, respectively. Apart from the apparent deviation at low EtOH concentration that might be mitigated by enlarging the simulation box or averaging over several trajectories, several studies focused on CO${}_{2}$ diffusion in brut-labeled champagnes. The experimental diffusion coefficients estimated in the present work are in very close agreement with previous ${}^{13}$C NMR experiments performed in carbonated hydroalcoholic solutions respecting brut-labeled champagnes proportions [33], although we are aware that there might be a bias in this comparison because the hydrodynamic radii used to get our experimental diffusion coefficients were derived from the same NMR measurements. The most recent theoretical CO${}_{2}$ diffusion coefficients obtained for these systems [34] are also in good agreement with our results unlike former results obtained with the TIP5P (Transferable Intermolecular Potential with 5 Points) and SPC/E water models [41,43] that sometimes significantly depart from the experimental curve.

**Figure 4:**
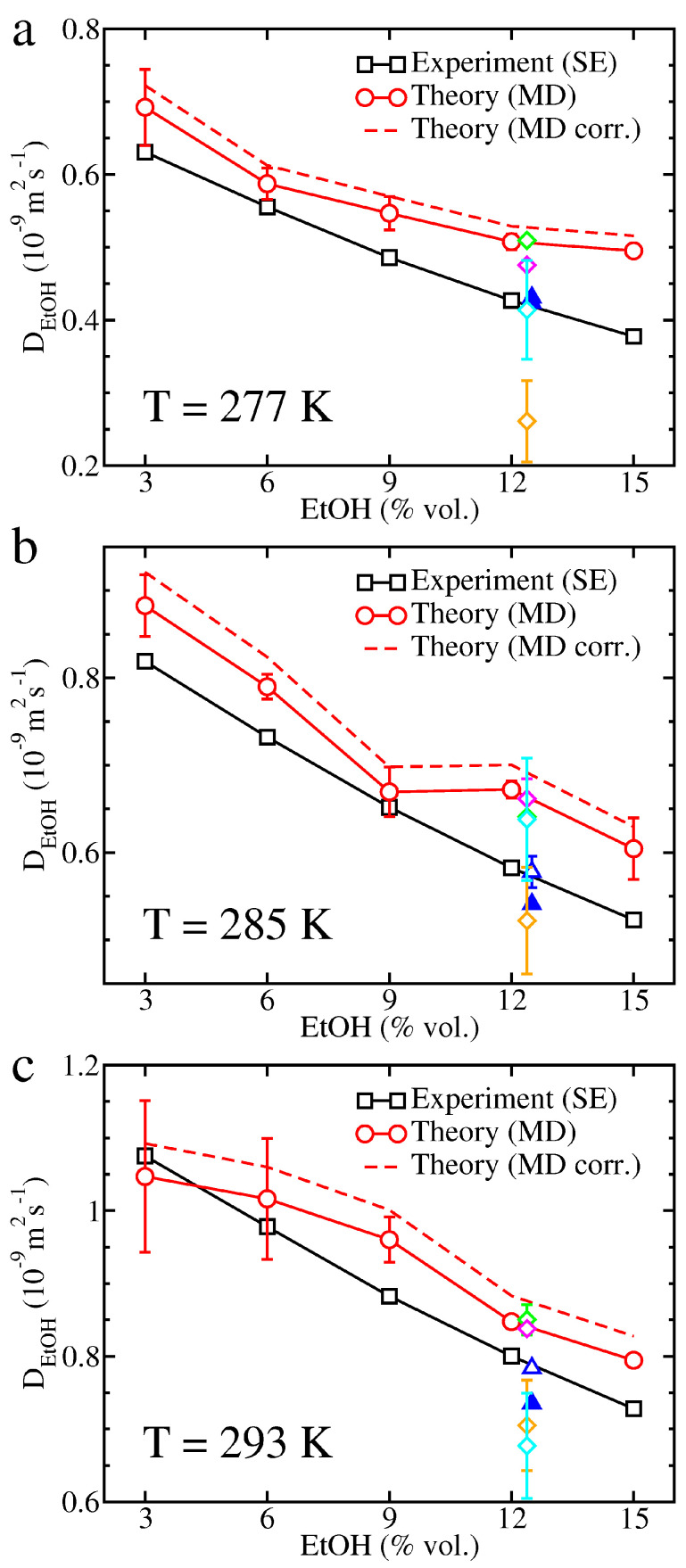
Experimental and theoretical EtOH diffusion coefficients in carbonated hydroalcoholic solutions at (**a**) *T* = 277 K, (**b**) *T* = 285 K, and (**c**) *T* = 293 K. The definition of symbols and colors is the same as in Figure 3.

Theoretical and experimental EtOH diffusion coefficients are illustrated in Figure 4. Experimental data remain in perfect agreement with former NMR measurements performed on carbonated hydroalcoholic solutions respecting brut-labeled champagnes proportions and EtOH diffusion coefficients in champagnes are once again lowered due to the multicomponent nature of these mixtures [33]. In contrast, theoretical results overestimate slightly experimental data and correcting EtOH diffusion coefficients for system-size dependence due to periodic boundary conditions degrades even more the agreement with experiments. These theoretical results are, however, in agreement with recent MD simulations carried out on carbonated hydroalcoholic mixture with an EtOH concentration of ∼12.4% vol. [34] and water molecules described by the TIP4P/2005 (Transferable Intermolecular Potential with 4 Points/2005) [44] and OPC models [40]. No clear trend emerge from former results of MD simulations achieved by using the SPC/E and TIP5P water models since they can underestimate, overestimate or even be in good agreement with experiments depending on temperature.

Finally, theoretical densities only overestimate experimental values by 1 to 4 kg m${}^{-3}$ at all temperatures and EtOH concentrations (see Tables S1 and S2 of the Supplementary Materials). The high quality of these theoretical densities contrasts with conclusions brought in a recent publication [34] where theoretical densities obtained with the OPC water model were found to underestimate experimental ones by ∼$6\;\mathrm{kg}\;m^{-3}$ at temperatures ranging from 277 K to 293K and an EtOH concentration of 12.4% [33]. This discrepancy comes from the use of different benchmark experimental densities, the set of measurements considered in the present work being of higher quality. Indeed, former experimental densities obtained for hydroalcoholic solutions extended from 990 kg m${}^{-3}$ to 993 kg m${}^{-3}$, namely about 8 kg m${}^{-3}$ above the expected values.

## 3. Discussion

### 3.1. Definitions of Hydrodynamic Radii

Experimental diffusion coefficients are determined by applying the Stokes-Einstein (SE) relation where the radii of CO${}_{2}$ and EtOH are former estimates based on ${}^{13}$C NMR measurements on carbonated hydroalcoholic solutions respecting brut-labeled champagnes concentrations [33]. Thus, it seems relevant to evaluate the sensitivity of experimental diffusion coefficients to the magnitude of these radii, all the more since variations in these diffusion coefficients may alter our conclusions when comparing with theoretical values extracted from MD simulations.

The SE relation is a mathematical formula primarily devised to evaluate the diffusion of a large spherical particle within a solvent composed of much smaller molecules where the Stokes’ law applies [45]. However, this formula was proved useful in other contexts provided that an effective radius could be guessed for the diffusing particle. In particular, it was found well suited to evaluate the diffusion of CO${}_{2}$ in multicomponent mixtures like champagnes [33,38,39]. Intuitively, we might approximate the hydrodynamic radius *R* of a species in a mixture as $R={\left(3V/4\pi \right)}^{1/3}$, where *V* would be the volume occupied by the species in the mixture but such a definition might be involved to apply in multicomponent liquids. Conversely, several simple structural radii can be defined for a molecule, like the rms distance of its atoms to its center of mass ($R_{rms}$) or its radius of gyration ($R_{gyr}$). In our MD simulations devoted to carbonated hydroalcoholic mixtures, these structural radii do not change when varying the temperature or alcoholic degree because bonds are constrained. For CO${}_{2}$, we found $R_{rms}\approx 0.94$ Å and $R_{gyr}\approx 0.98$ Å, values alike those derived from other CO${}_{2}$ models, like TraPPE (Transferable Potentials for Phase Equilibria) [46] or MSM-ZD [34,47], or from the CO${}_{2}$ structure available in the NIST database [48], namely $R_{rms}\approx 0.95$ Å and $R_{gyr}\approx 0.99$ Å. For EtOH, we found $R_{rms}\approx 1.58$ Å and $R_{gyr}\approx 1.19$ Å in agreement with values deduced from the EtOH structure available in the NIST database, namely $R_{rms}\approx 1.58$ Å and $R_{gyr}\approx 1.18$ Å. These radii can also be compared with the NMR-based radii used to determine the experimental diffusion coefficients, namely $R_{{\mathrm{CO}}_{2}}=0.95-1.05$ Å and $R_{\mathrm{EtOH}}=1.80-1.88$ Å at temperatures ranging from 277 K to 293 K and EtOH concentrations of ∼12.5%vol. [33]. Both structural radii $R_{rms}$ and $R_{gyr}$ provide a proper estimate of the NMR-based radii for CO${}_{2}$ but they underestimate strongly the experimental values for EtOH and replacing the NMR-based radii by $R_{rms}$ or $R_{gyr}$ to plot experimental data reported in Figure 3 and Figure 4 can have an influence on the comparisons between theory and experiment.

We have previously noticed that, apart from some intriguing deviation at T=293K and low EtOH concentrations, theoretical and experimental CO${}_{2}$ diffusion coefficients were in good agreement with each other. Moreover, replacing the NMR-based radii by the CO${}_{2}$ rms radius or radius of gyration to calculate the experimental CO${}_{2}$ diffusion coefficients would not significantly alter this conclusion since the deviation between all these radii does not exceed 10%. Experimental diffusion coefficients would be somewhat lower at T=277K with $R_{gyr}$ and higher at T=285K and T=293K in both cases which would degrade the agreement at low EtOH concentrations but improve it at the three highest EtOH concentrations at T=285K (see Figure S1 of the Supplementary Materials). In contrast, both structural definitions of radii underestimate the experimental radius for EtOH, the discrepancy growing to ∼14–17% for $R_{rms}$ and 51–55% for $R_{gyr}$. Replacing the NMR-based radii by $R_{rms}$ to determine the experimental EtOH diffusion coefficients would improve the agreement at T=277K but theoretical EtOH diffusion coefficients would tend to underestimate slightly experimental values and using $R_{gyr}$ instead of $R_{rms}$ would increase even more this underestimation (see Figure S2 of the Supplementary Materials). Moreover, the experimental EtOH diffusion coefficients would then differ from NMR data. Obviously, no structural definition is perfectly suited to model non-spherical molecules like CO${}_{2}$ or EtOH, and we can at most expect that the volume occupied by the molecule is fairly reproduced. However, in absence of exhaustive tables of experimental radii, $R_{rms}$ appears to be a more accurate definition for the effective radius to be used in the SE relation than $R_{gyr}$ as pointed out in a previous work [34]. This comes from the mass-weighting of atomic positions in the definition of the radius of gyration that gives, for instance, too much weight to the central carbon atoms at the expense of the peripheral hydrogen atoms in the case of EtOH.

The most appropriate way to unravel the mystery behind the partial disagreement between theory and experiment would probably be to compare our experimental results with NMR data on carbonated hydroalcoholic solutions at several temperatures and alcoholic degrees, data that are unfortunately unavailable to the best of our knowledge. This would enable us to evaluate the accuracy of former NMR-based radii in predicting the diffusion coefficients of CO${}_{2}$ and EtOH at several ethanol concentrations and temperatures, then getting more accurate results to compare with realistic alcoholic sparkling beverages.

### 3.2. Possible Improvements of the Theoretical Approach

Beside the question on the definition of an optimal hydrodynamic radius representative of non-spherical molecules in carbonated hydroalcoholic beverages, the quality of theoretical results derived from MD simulations might also be improved by averaging the statistical data over additional molecules or longer times, employing a more sophisticated approach to evaluate diffusion coefficients, or optimizing the parameters of the molecular models. Although increasing the system size and simulation time could mechanically improve the accuracy of diffusion coefficients, at the expense of additional computational cost, we do not expect significant improvements of the results because equilibrium was reached after 20 ns, statistical uncertainties and corrections for system-size dependence are already included in our calculations, and diffusion coefficients of CO${}_{2}$, the species less abundant for which averages should be the least accurate, are already of good quality when compared with experiments.

Maxwell-Stefan [49] or Fick [50] multicomponent diffusion coefficients could also be evaluated without assuming low concentrations of some species in the mixture, as done by Garcia-Ratés et al. to describe CO${}_{2}$ diffusion in brines [51], although this approach is best suited for high concentrations of solutes. As an example, Garcia-Ratés et al. performed MD simulations in boxes containing ∼3000 water molecules, up to 120 CO${}_{2}$ molecules and 216 monatomic ions (Na${}^{+}$ or Cl${}^{-}$). Our simulations contain $4\times 10^{4}$ water molecules, 200 CO${}_{2}$ molecules, and up to 2199 EtOH molecules, which suggests that CO${}_{2}$ can probably be considered as traces in the mixture, but this assumption is more questionable for EtOH when its abundance reaches several thousands of molecules. It is, however, worth noting that the worse agreements with experiments occurs at low ethanol levels rather than high ones (see Figure 4) which would suggest that our approach to evaluate EtOH diffusion coefficient is not the main source of discrepancy, even at the higher EtOH concentrations. The accuracy of our theoretical method should, therefore, mainly rely on the efficiency of the molecular models.

In a recent study, Ahmed Khaireh et al. have analyzed the effect of six water models and three CO${}_{2}$ models, by constraining bonds or leaving them free, on CO${}_{2}$ diffusion in carbonated hydroalcoholic mixtures respecting brut-labeled champagne proportions as a function of temperature [34]. CO${}_{2}$ diffusion coefficients were derived from MSDs and the integration of velocity autocorrelation functions. They concluded that the most accurate CO${}_{2}$ diffusion coefficients were obtained for the OPC water model, and to a lesser extent the TIP4P/2005 water model, with little influence of the CO${}_{2}$ model in use. By following the same approach we confirmed the quality of CO${}_{2}$ diffusion coefficients in carbonated hydroalcoholic mixtures, especially at EtOH concentrations in between 6 and 15% vol. (see Figure 3). However, Ahmed Khaireh et al. only considered the OPLS-aa (Optimized Potentials for Liquid Simulations-all atom) force field to parameterize EtOH and testing other force fields, like CHARMM (Chemistry at HARvard Macromolecular Mechanics) [52], might improve EtOH diffusion coefficients. In particular, studies using CHARMM had been undertaken on brut-labeled champagnes with the SPC/E and TIP5P water models [32,33]. Although the TIP5P model yielded an overestimation of the CO${}_{2}$ diffusion coefficients, these coefficients were properly described at the higher temperatures (T≳285K) with the SPC/E model. Conversely, the TIP5P water model lead somewhat better agreement with experimental EtOH diffusion coefficients than the SPC/E water model. Combining the CHARMM force field with the OPC water model and the EPM2 CO${}_{2}$ model might be an opening of the present work, although we must note that OPLS-aa should be a choice force field to describe EtOH since this molecule is natively parameterized in OPLS-aa.

It is also worth noting that molecular mechanics is probably more suited to reproduce the properties of rigid nonpolar CO${}_{2}$ molecules than the properties of the more flexible EtOH molecules, which would be better modeled by quantum-mechanical approaches. At the molecular mechanics level of accuracy, Perret at al. [32] estimated the number of hydrogen bonds (H bonds) in carbonated hydroalcoholic solutions representative of brut-labeled champagnes by assuming a H bond is formed between a hydrogen atom and an oxygen atom if the O${}_{d}$-O${}_{a}$ distance between the donor oxygen atom O${}_{d}$ and the acceptor oxygen atom O${}_{a}$ remains below 0.35 nm and the H-O${}_{d}$-O${}_{a}$ angle does not exceed 35°, as advocated by Chandler [53]. From this definition of H bonds, they found that EtOH created roughly fifty times more H bonds than CO${}_{2}$ when water molecules were described by the SPC/E and TIP5P models. This result, later confirmed by Lv et al. [54], contributed to account for the smallness of EtOH diffusion coefficients compared with CO${}_{2}$ ones. Although it is hard to predict how an accurate description of the rotation of EtOH methyl groups would precisely alter the number of H bonds involving EtOH molecules, we may expect an increase of this number. In such a case, the mobility of these molecules, and subsequently the values of their diffusion coefficients, might decrease which should improve the agreement with experiments.

## 4. Conclusions

The analysis of transport phenomena in sparkling beverages, among which CO${}_{2}$ and EtOH diffusions occupy a choice position, is essential to better comprehend the mechanisms behind the formation and growth of CO${}_{2}$ bubbles and the release of organoleptic compounds at the free surface of the liquid. In the present study, MD simulations carried out on carbonated hydroalcoholic mixtures at three temperatures and six alcoholic degrees were proved useful to predict CO${}_{2}$ diffusion coefficients, whereas EtOH diffusion coefficients seemed somewhat overestimated. Disagreements with experiments are mainly attributed to limitations of the molecular models, especially that of EtOH, although an increase of the statistics and improvements of the method to evaluate diffusion coefficients might also influence the results. However, this conclusion was drawn on the basis of experimental diffusion coefficients derived from the Stokes-Einstein relation. The values of these diffusion coefficients, therefore, depend on the accuracy of viscosity measurements and on the definition of the effective radii used for the diffusing species (CO${}_{2}$ and EtOH here). This observation made us assert that accurate ${}^{13}C$ NMR measurements at several temperatures and alcoholic degrees would be recommended to confirm the quality of experimental diffusion coefficients reported in our study and, equivalently, ensure the suitability of the molecular radii inserted in the SE relation to get them.

Despite possible inaccuracies from both the experimental and theoretical sides, this comprehensive investigation of the influence of the alcoholic degree on CO${}_{2}$ and EtOH diffusion at several temperatures, together with a recent work focused on the effect of water and CO${}_{2}$ models on CO${}_{2}$ diffusion coefficients in carbonated hydroalcoholic solutions respecting brut-labeled champagnes proportions [34], open new avenues for theoretical studies on sparkling beverages. First of all, they can serve as reference data to evaluate the influence of other species, like sugars, on CO${}_{2}$ diffusion. As an example, champagnes are mainly composed of water, CO${}_{2}$, and EtOH but a more sophisticated model would include glycerol, tartaric and lactic acids, and various amounts of sugar [38] to evaluate how the degassing of CO${}_{2}$ is influenced by the concentration of sugar. CO${}_{2}$ bubbles, whose size depends on gas exchanges at the interface between the bubble and bulk champagne, are also known to take organoleptic compounds with them during their ascent to the free surface of the liquid, and their subsequent explosion releases a cloud of droplets with a composition slightly different from the bulk one [12]. Nanometer-sized hydroalcoholic droplets could be first designed to tackle the evaporation dynamics of small droplets and compare the corresponding results with available experimental data [13], before making the droplet bigger and its composition closer to that of aerosols representative of sparkling wines. Finally, a proper molecular modeling of sparkling beverages is needed to investigate the diffusion of CO${}_{2}$ through the walls of cellulose fibers, nucleation sites responsible for the formation of CO${}_{2}$ bubbles [55]. The present work is, therefore, an additional brick laid to better understand the influence of molecular models on CO${}_{2}$ and EtOH diffusion in sparkling beverages that calls for future theoretical studies in this field of research.

## 5. Materials and Methods

### 5.1. Molecular Dynamics Simulations

**Figure 5:**
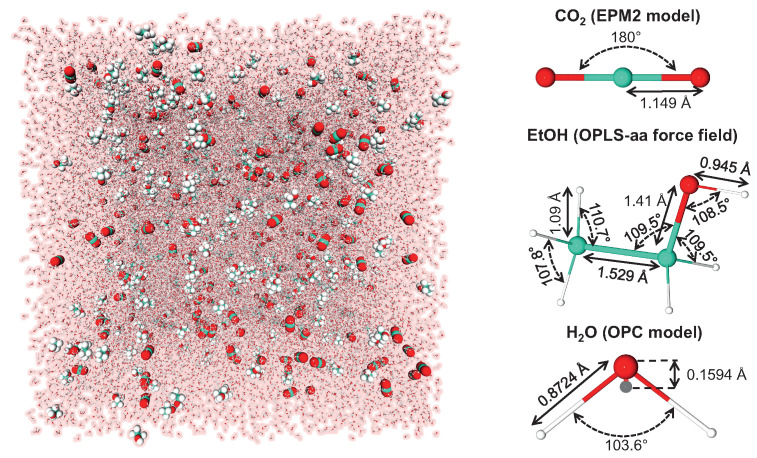
Example of simulation box containing $4\times 10^{4}$ H${}_{2}$O, 200 CO${}_{2}$ and 385 EtOH (3% vol.). Bond distances and angles of the three molecules used in our model are indicated on the right panel. Pictures were generated with the VMD (Visual Molecular Dynamics) and Protein Imager softwares [62,63].

A total of 18 MD simulations were carried out with the open-source massively parallel GROMACS software (2018 versions) [56,57,58,59] on carbonated hydroalcoholic solutions at six ethanol (EtOH) concentrations and three temperatures relevant for sparkling alcoholic beverage applications, namely 277 K (fridge temperature), 285 K (cellar temperature), and 293 K (ambient temperature). A cubic box, containing $4\times 10^{4}$ water molecules described by the OPC (Optimal Point Charge) 4-point rigid water model [40], 200 CO${}_{2}$ molecules described by the popular EPM2 (Elementary Physical Model 2) model [60], and a variable number *N* of EtOH molecules described by the OPLS-aa (Optimized Potentials for Liquid Simulations-all atom) force field [61] was built with periodic boundary conditions imposed in the three spatial directions, as illustrated in Figure 5.

An alike simulation box has been recently employed by Ahmed Khaireh et al. to investigate CO${}_{2}$ diffusion in brut-labeled champagnes (alcoholic degree of ∼12.4%vol.) as a function of temperature [34]. *N* is calculated for water and EtOH properties at T=285K, temperature at which wines undergo their second fermentation.

(2) $$N=N_{H_{2}O}\frac{\rho_{\mathrm{EtOH}}M_{H_{2}O}}{\rho_{H_{2}O}\left(r-1\right)M_{\mathrm{EtOH}}},$$

where $N_{H_{2}O}=4\times 10^{4}$, $\rho_{H_{2}O}=999.49\;\mathrm{kg}\;m^{-3}$, $\rho_{\mathrm{EtOH}}=796.06\;\mathrm{kg}\;m^{-3}$, $M_{i}$ is the molar mass of species *i*, and $r=V_{tot}/V$ is the ratio between the total volume of the mixture and that occupied by EtOH. Table 1 reports the values of *N* for alcoholic degrees ranging from 0 to 15% vol. by steps of 3% vol. Lennard-Jones and van der Waals interactions are truncated at a distance of 1.5nm, a smooth particle-mesh Ewald (SPME) summation technique is applied to handle long-range electrostatic interactions [64,65,66], and bonds are constrained during the simulations. The whole range of temperatures and EtOH abundances spanned during our simulations only yield a 5% increase of the box side length *L* from ∼10.6nm to ∼11.2nm.

For each MD simulation, the system is first equilibrated for 1 ns in the NVT ensemble by fixing the appropriate temperature and density. A subsequent 19-ns equilibration run is performed in the NPT ensemble at a pressure of 1 bar and followed by a 10-ns production run devoted to the storage of statistical data, atomic positions, and velocities every 1 ps. Temperature is controlled by applying a Nosé-Hoover thermostat with a time constant of 0.5 ps [67,68] and pressure is maintained by a Parinello-Rahman barostat with a time constant of 0.2 ps [69]. Carbonated hydroalcoholic solutions being mainly composed of water, their isothermal compressibility is taken as that of pure water, namely $\kappa \left(277\;K\right)=4.95\times 10^{-5}\;{\mathrm{bar}}^{-1}$, $\kappa \left(285\;K\right)=4.73\times 10^{-5}\;{\mathrm{bar}}^{-1}$, and $\kappa \left(293\;K\right)=4.59\times 10^{-5}\;{\mathrm{bar}}^{-1}$ [34,70,71].

### 5.2. Theoretical Diffusion Coefficients

Carbonated hydroalcoholic solutions, and more generally sparkling beverages, are multicomponent systems and the diffusion coefficients of solvated species should be evaluated by solving the generalized Fick equations [50,72] or alike systems of equations based on Maxwell-Stefan or Onsager theories [49,73,74]. However, recent works on brut-labeled champagnes have shown that the properties of these mixtures (e.g., water representing 95% of the quantity of matter, homogeneity and isotropy of the liquid on average, no chemical reaction on short time scales) make them properly modeled by a Fick’s law very similar to that specific to simple fluids or binary liquids [32]. The probability density of diffusing species is, therefore, gaussian and the diffusion coefficients *D* of CO${}_{2}$ and EtOH can be estimated by computing their mean-squared displacement (MSD) since $MSD\left(t\right)=\langle {\left[\vec{r}\left(t\right)-\vec{r}\left(0\right)\right]}^{2}\rangle =6Dt$ at long times. Each MSD is averaged over the number of diffusing molecules (i.e., 200 for CO${}_{2}$ and up to 2199 for EtOH) and $10^{4}$ time origins representative of our data storage frequency [32]. However, it is worth noting that diffusion coefficients computed from MD simulations with stick periodic boundary conditions should be corrected because the results can depend on the system size as pointed out by Yeh and Hummer [75]. Typical corrections depend on the viscosity of the liquid (η) but, by applying the SE relation, we can find a formula for the corrected diffusion coefficient ($D_{0}$) that only depends on the original diffusion coefficient ($D_{PBC}$), the simulation box side length (*L*), and the hydrodynamic radius of the species (*R*).

(3) $$D_{0}=D_{PBC}+\frac{\xi k_{B}T}{6\pi \eta L}\approx D_{PBC}{\left(1-\frac{\xi R}{L}\right)}^{-1},$$

where $k_{B}$ is the Boltzmann contant, *T* is the temperature, and ξ=2.837297 is a constant [75]. Moreover, comparisons with ${}^{13}$C NMR spectroscopic measurements carried out on carbonated hydroalcoholic solutions respecting brut-labeled champagnes proportions have shown that the dynamic viscosity of these mixtures could be properly predicted by replacing the hydrodynamic radius by the root-mean-square (rms) atomic distance of a diffusing species like CO${}_{2}$ or EtOH [33,34]. The same definition of *R*, easily extracted from MD simulations, is, therefore, chosen in the present work to calculate the corrections brought to theoretical CO${}_{2}$ and EtOH diffusion coefficients.

### 5.3. Viscometry and Experimental Diffusion Coefficients

A rolling ball viscometer, Lovis 2000 M/ME (Anton Paar, Austria), was used to determine the dynamic viscosity of hydroalcoholic solutions (with alcoholic degrees ranging from 0 to 15% vol.). This equipment measures the rolling time of a ball through liquids according to Hoeppler’s falling ball principle [76]. The samples were measured with a steel ball and a 1.59 mm diameter capillary (0.3–90mPa·s), at three temperatures: *T* = 277 K, 285 K, and 293 K. The repeatability in the viscosity measurement is up to 0.1%, and its reproducibility is up to 0.5%, according to the manufacturer [76]. The required values of density for each hydroalcoholic solution were obtained from the literature [77].

Experimental CO${}_{2}$ and EtOH diffusion coefficients are derived from viscosity measurements by applying the SE relation

(4) $$D=\frac{k_{B}T}{6\pi \eta R},$$

where $k_{B}$ is the Boltzmann constant, *T* is the temperature (in K), η is the viscosity (in Pa·s), and *R* is the hydrodynamic radius of the species under consideration (in m). To the best of our knowledge, there is no extensive data set in the literature on CO${}_{2}$ and EtOH hydrodynamic radii as a function of temperature and alcoholic degree. However, these hydrodynamic radii have been evaluated as a function of temperature for carbonated hydroalcoholic mixtures with a concentration of ethanol representative of brut-labeled champagnes [33]. They were derived from Equation (4) by using diffusion coefficients determined from ${}^{13}$C NMR measurements and viscosities measured with a similar experimental setup as in the present study. For carbon dioxide, hydrodynamic radii were $R_{{\mathrm{CO}}_{2}}\left(277\;K\right)=0.95$ Å, $R_{{\mathrm{CO}}_{2}}\left(285\;K\right)=1.03$ Å, and $R_{{\mathrm{CO}}_{2}}\left(293\;K\right)=1.03$ Å. For ethanol, they were $R_{\mathrm{EtOH}}\left(277\;K\right)=1.81$ Å, $R_{\mathrm{EtOH}}\left(285\;K\right)=1.85$ Å, and $R_{\mathrm{EtOH}}\left(293\;K\right)=1.80$ Å. These experimental hydrodynamic radii will be used as reference values to evaluate experimental CO${}_{2}$ and EtOH diffusion coefficients at all alcoholic degrees and compared with theoretical radii extracted from MD simulations.

## Figures and Tables

**Table 1:** Number of EtOH molecules (*N*) in simulation boxes composed of $4\times 10^{4}$ water molecules and 200 CO${}_{2}$ molecules for EtOH levels ranging from 0 to 15% vol. *N* values are computed at a reference temperature of 285 K.

EtOH (% vol.)	0	3	6	9	12	15
N	0	385	795	1232	1699	2199

## Data Availability

Some data presented in this study are available in the Supplementary Materials. The other data presented in this study are available on request from the corresponding authors. These data are not publicly available due to their storage on institutional data center facilities with i/o restrictions.

## Supplementary Materials

The following are available online, Table S1: Experimental densities ($\rho_{exp}$) and viscosities ($\eta_{exp}$) of hydroalcoholic mixtures at three temperatures and six alcoholic degrees, and corresponding CO${}_{2}$ and EtOH diffusion coefficients ($D_{{\mathrm{CO}}_{2}}^{exp}$ and $D_{\mathrm{EtOH}}^{exp}$) derived from the Stokes-Einstein relation and NMR-based radii, Table S2: Average pressures 〈P〉 and densities 〈ρ〉 derived from MD simulations, as well as diffusion coefficients of species *i* deduced from MSDs ($D_{i}$) and diffusion coefficients corrected for size-system dependence ($D_{i}^{0}$) at three temperatures and six alcoholic degrees, Figure S1: Experimental and theoretical CO${}_{2}$ diffusion coefficients in carbonated hydroalcoholic solutions at three temperatures and six alcoholic degrees (influence of $R_{rms}$ and $R_{gyr}$ on the results), Figure S2: Experimental and theoretical EtOH diffusion coefficients in carbonated hydroalcoholic solutions at three temperatures and six alcoholic degrees (influence of $R_{rms}$ and $R_{gyr}$ on the results).

## Abbreviations

The following abbreviations are used in this manuscript:

CHARMM	Chemistry at HARvard Macromolecular Mechanics
EPM2	Elementary Physical Model 2
MD	Molecular Dynamics
MSD	Mean-Squared Displacement
MSM-ZD	Murthy, Singer, and McDonald—Zhang and Duan
NIST	National Institute of Standards and Technology
NMR	Nuclear Magnetic Resonance
OPC	Optimal Point Charge
OPLS-aa	Optimized Potentials for Liquid Simulations-all atom
RMS	Root Mean Square
SPC/E	Extended Simple Point Charge
TIP4P/2005	Transferable Intermolecular Potential with 4 Points/2005
TIP5P	Transferable Intermolecular Potential with 5 Points
TraPPE	Transferable Potentials for Phase Equilibria
