# Supplementary material for: Unveiling Carbon Dioxide and Ethanol Diffusion in Carbonated Water-Ethanol Mixtures by Molecular Dynamics Simulations
Source: Molecules. 2021 Mar 19;26(6):1711. doi: 10.3390/molecules26061711 (PMC8003404; doi:10.3390/molecules26061711)
Supplement: Supplementary file 1 [file molecules-26-01711-s001.pdf]

Unveiling Carbon Dioxide and Ethanol Diffusion in Carbonated Water-Ethanol Mixtures by Molecular Dynamics Simulations (supplementary materials)

Mohamed Ahmed Khaireh, Marie Angot, Clara Cilindre, Gérard Liger-Belair,* and David A. Bonhommeau*

Université de Reims Champagne Ardenne, CNRS, GSMA UMR 7331, 51097 Reims, France.

E-mail: gerard.liger-belair@univ-reims.fr; david.bonhommeau@univ-reims.fr

Fax: +033 (0)326 913147

*To whom correspondence should be addressed

Table S1: Experimental densities (ρ_{exp}) and viscosities (η_{exp}) of hydroalcoholic mixtures at three temperatures and six alcoholic degrees, and corresponding CO_2 and EtOH diffusion coefficients ($D^{exp}_{CO_2}$ and D^{exp}_{EtOH}) derived from the Stokes-Einstein relation and NMR-based radii.

T (K)	EtOH (% vol.)	ρ_{exp} ($kg\,m^{-3}$)	η_{exp} ($\times10^{-3}$ Pa · s)	$D^{exp}_{CO_2}$ ($\times10^{-9}\,m^2\,s^{-1}$)	D^{exp}_{EtOH} ($\times10^{-9}\,m^2\,s^{-1}$)
277	0	999.57	1.566 ± 0.008	1.36 ± 0.01	—
	3	995.19	1.777 ± 0.009	1.20 ± 0.01	0.63 ± 0.01
	6	991.27	2.018 ± 0.011	1.06 ± 0.01	0.56 ± 0.01
	9	987.75	2.307 ± 0.012	0.93 ± 0.01	0.49 ± 0.01
	12	984.63	2.625 ± 0.014	0.81 ± 0.01	0.43 ± 0.01
	15	981.83	2.970 ± 0.015	0.72 ± 0.01	0.38 ± 0.01
285	0	999.29	1.235 ± 0.007	1.64 ± 0.01	—
	3	994.90	1.378 ± 0.007	1.47 ± 0.01	0.82 ± 0.01
	6	990.90	1.541 ± 0.008	1.32 ± 0.01	0.73 ± 0.01
	9	987.24	1.731 ± 0.009	1.17 ± 0.01	0.65 ± 0.01
	12	983.88	1.937 ± 0.010	1.05 ± 0.01	0.58 ± 0.01
	15	980.77	2.158 ± 0.011	0.94 ± 0.01	0.52 ± 0.01
293	0	998.20	1.009 ± 0.006	2.07 ± 0.02	—
	3	993.81	1.109 ± 0.006	1.88 ± 0.01	1.08 ± 0.01
	6	989.73	1.219 ± 0.007	1.71 ± 0.01	0.98 ± 0.01
	9	985.92	1.351 ± 0.007	1.54 ± 0.01	0.88 ± 0.01
	12	982.35	1.490 ± 0.008	1.40 ± 0.01	0.80 ± 0.01
	15	978.97	1.638 ± 0.009	1.27 ± 0.01	0.73 ± 0.01

Table S2: Average pressures $\langle P \rangle$ and densities $\langle \rho \rangle$ derived from MD simulations as well as diffusion coefficients of species i deduced from MSDs (D_i) and diffusion coefficients corrected for size-system dependence (D_i^0) at three temperatures and six alcoholic degrees. The average temperature extracted from MD simulations is not indicated because it never deviates from the target temperature by more than 0.01 K.

T (K)	EtOH (% vol)	$\langle P \rangle$ (bar)	$\langle \rho \rangle$ (kg m^{-3})	D_{CO_2} ($\times 10^{-9}$ m^2 s^{-1})	$D^0_{CO_2}$ ($\times 10^{-9}$ m^2 s^{-1})	D_{EtOH} ($\times 10^{-9}$ m^2 s^{-1})	D^0_{EtOH} ($\times 10^{-9}$ m^2 s^{-1})
277	0	1.30 ± 0.08	1003.70 ± 0.03	1.24 ± 0.18	1.27	—	—
	3	1.17 ± 0.05	999.03 ± 0.02	0.99 ± 0.08	1.01	0.69 ± 0.06	0.72
	6	1.21 ± 0.10	994.81 ± 0.03	1.02 ± 0.03	1.04	0.59 ± 0.03	0.61
	9	1.02 ± 0.05	991.03 ± 0.03	0.95 ± 0.01	0.97	0.55 ± 0.03	0.57
	12	1.15 ± 0.08	987.51 ± 0.03	0.92 ± 0.09	0.94	0.51 ± 0.02	0.53
	15	1.10 ± 0.06	984.24 ± 0.02	0.80 ± 0.01	0.82	0.50 ± 0.01	0.52
285	0	1.24 ± 0.05	1002.53 ± 0.01	1.48 ± 0.14	1.52	—	—
	3	1.18 ± 0.06	997.86 ± 0.04	1.40 ± 0.10	1.44	0.88 ± 0.04	0.92
	6	1.09 ± 0.12	993.55 ± 0.02	1.14 ± 0.01	1.17	0.79 ± 0.02	0.82
	9	1.21 ± 0.09	989.56 ± 0.02	1.30 ± 0.02	1.33	0.67 ± 0.03	0.70
	12	1.16 ± 0.06	985.85 ± 0.01	1.09 ± 0.08	1.12	0.67 ± 0.01	0.70
	15	1.18 ± 0.04	982.31 ± 0.03	1.03 ± 0.04	1.06	0.60 ± 0.04	0.63
293	0	1.23 ± 0.09	1000.82 ± 0.02	1.65 ± 0.16	1.69	—	—
	3	1.39 ± 0.08	996.05 ± 0.02	1.54 ± 0.11	1.58	1.05 ± 0.11	1.09
	6	1.15 ± 0.07	991.65 ± 0.03	1.61 ± 0.08	1.65	1.02 ± 0.09	1.06
	9	1.10 ± 0.04	987.48 ± 0.02	1.41 ± 0.30	1.45	0.96 ± 0.04	1.00
	12	1.19 ± 0.03	983.54 ± 0.02	1.41 ± 0.16	1.45	0.85 ± 0.01	0.88
	15	1.18 ± 0.06	979.78 ± 0.01	1.23 ± 0.23	1.26	0.79 ± 0.01	0.83

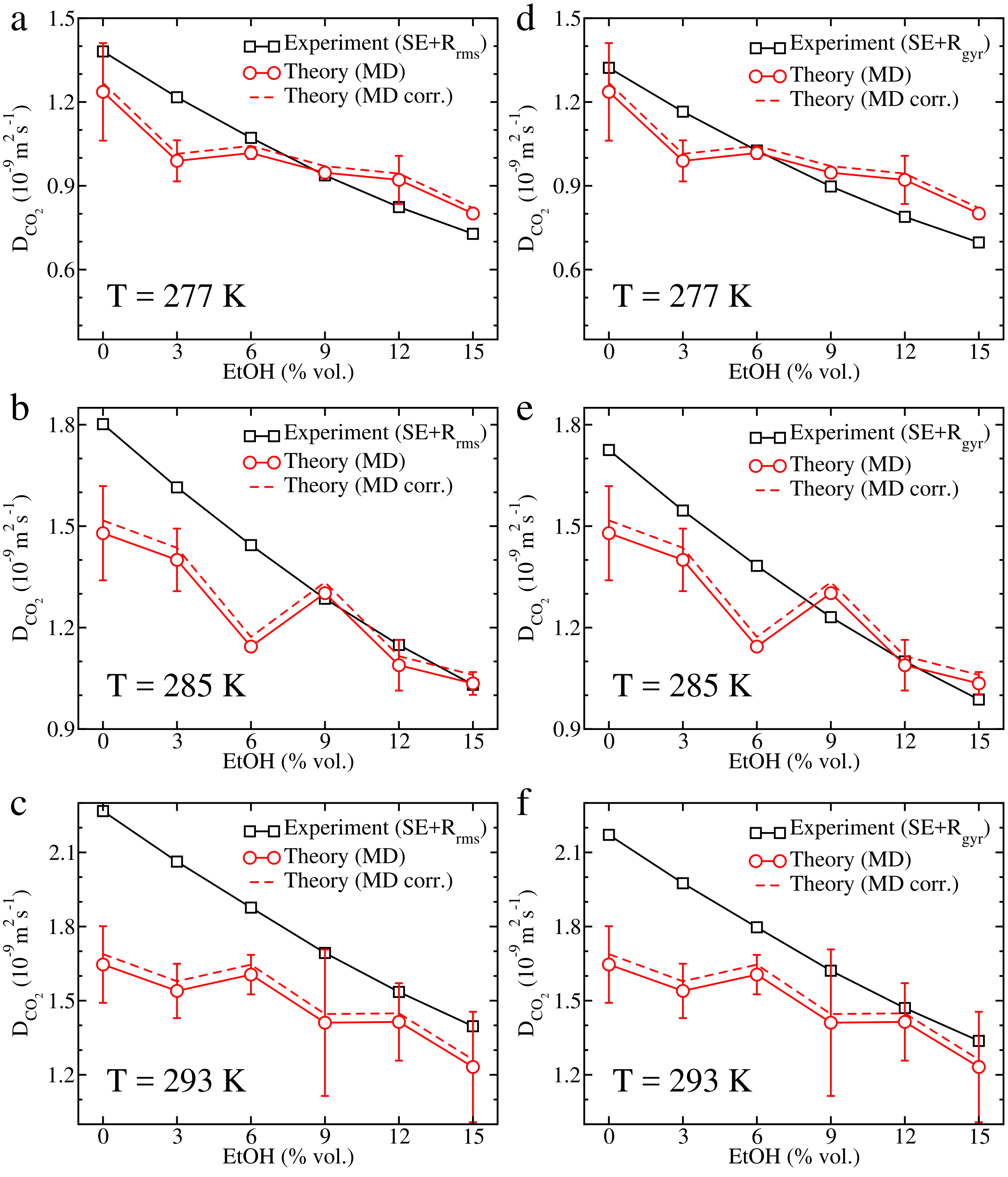

Figure S1: Experimental and theoretical CO_2 diffusion coefficients in carbonated hydroalcoholic solutions at three temperatures and six alcoholic degrees. Experimental values (black squares) are derived from the Stokes-Einstein relation (SE) by using CO_2 rms radii (R_{rms}) or radii of gyration (R_{gyr}) from MD simulations. (**a**) R_{rms} at $T = 277$ K, (**b**) R_{rms} at $T = 285$ K, and (**c**) R_{rms} at $T = 293$ K, (**d**) R_{gyr} at $T = 277$ K, (**e**) R_{gyr} at $T = 285$ K, and (**f**) R_{gyr} at $T = 293$ K. Theoretical values deduced from MD simulations (red circles) are reported together with theoretical diffusion coefficients corrected for system-size dependence (red dashed curve).

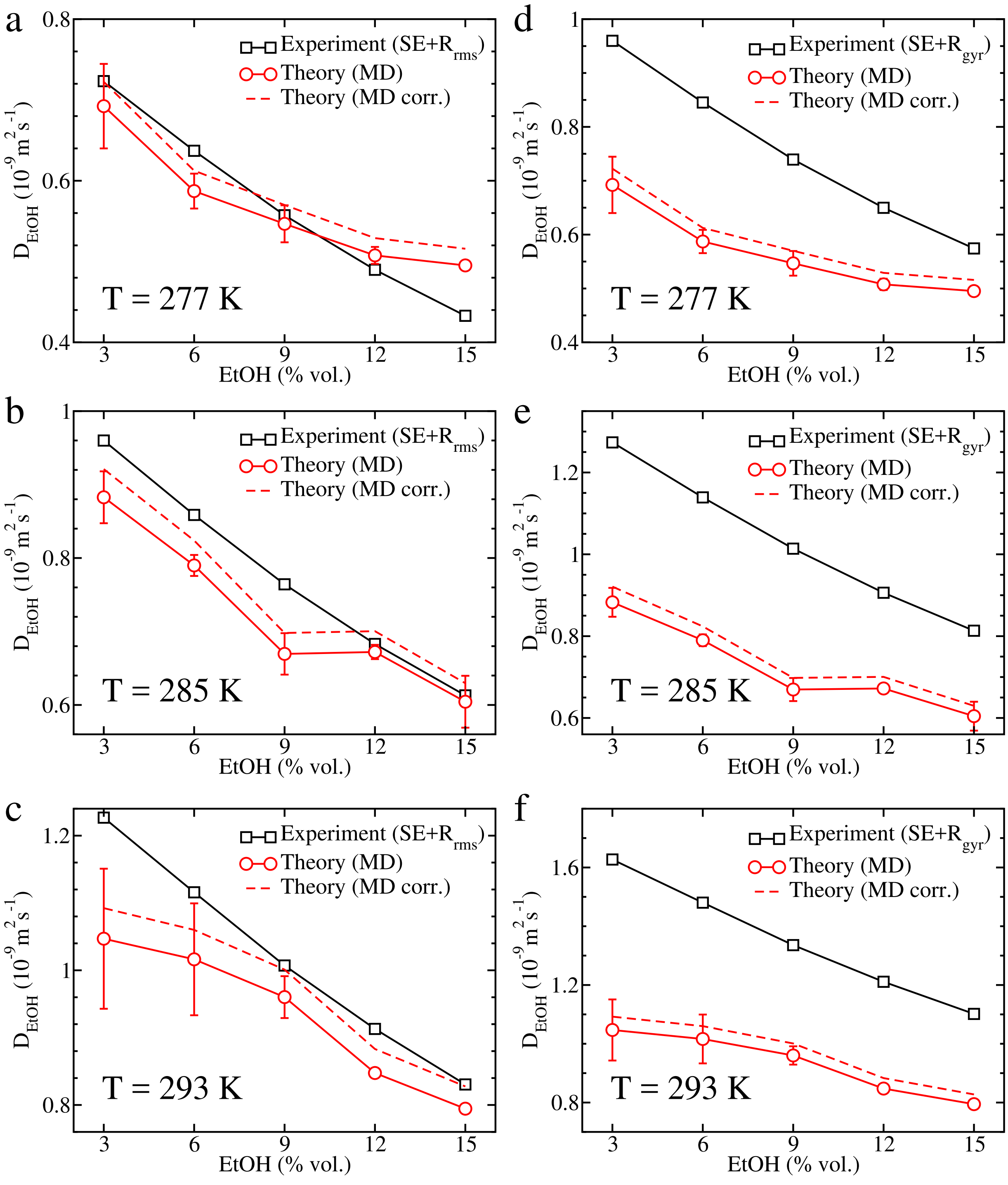

Figure S2: Experimental and theoretical EtOH diffusion coefficients in carbonated hydroalcoholic solutions at three temperatures and six alcoholic degrees. Experimental values (black squares) are derived from the Stokes-Einstein relation (SE) by using EtOH rms radii (R_{rms}) or radii of gyration (R_{gyr}) from MD simulations. (**a**) R_{rms} at $T = 277$ K, (**b**) R_{rms} at $T = 285$ K, and (**c**) R_{rms} at $T = 293$ K, (**d**) R_{gyr} at $T = 277$ K, (**e**) R_{gyr} at $T = 285$ K, and (**f**) R_{gyr} at $T = 293$ K. Theoretical values deduced from MD simulations (red circles) are reported together with theoretical diffusion coefficients corrected for system-size dependence (red dashed curve).
